# Using food network analysis to understand meal patterns in pregnant women with high and low diet quality

**DOI:** 10.1186/s12966-021-01172-1

**Published:** 2021-07-23

**Authors:** Carolina Schwedhelm, Leah M. Lipsky, Grace E. Shearrer, Grace M. Betts, Aiyi Liu, Khalid Iqbal, Myles S. Faith, Tonja R. Nansel

**Affiliations:** 1 Social and Behavioral Sciences Branch, Division of Intramural Population Health Research, Eunice Kennedy Shriver National Institute of Child Health and Human Development, National Institutes of Health, Bethesda, MD USA; 2 Present address: Max-Delbrueck-Center for Molecular Medicine in the Helmholtz Association (MDC), Molecular Epidemiology Research Group, Robert-Rössle-Straße 10, 13125 Berlin, Germany; 3 Department of Nutrition, Gillings School of Global Public Health, University of North Carolina Chapel Hill, Chapel Hill, NC USA; 4 Biostatistics and Bioinformatics Branch, Division of Intramural Population Health Research, Eunice Kennedy Shriver National Institute of Child Health and Human Development, National Institutes of Health, Bethesda, MD USA; 5 Department of Human Nutrition, Institute of Basic Medical Sciences, Khyber Medical University, Peshawar, Pakistan; 6 Department of Counseling, School and Educational Psychology, University at Buffalo Graduate School of Education, Buffalo, NY USA

**Keywords:** Meals, Breakfast, Lunch, Dinner, Snacks, Network analysis, Gaussian graphical models, Diet quality, Healthy eating index, Pregnancy

## Abstract

**Background:**

Little is known about how meal-specific food intake contributes to overall diet quality during pregnancy, which is related to numerous maternal and child health outcomes. Food networks are probabilistic graphs using partial correlations to identify relationships among food groups in dietary intake data, and can be analyzed at the meal level. This study investigated food networks across meals in pregnant women and explored differences by overall diet quality classification.

**Methods:**

Women were asked to complete three 24-h dietary recalls throughout pregnancy (*n* = 365) within a prospective cohort study in the US. Pregnancy diet quality was evaluated using the Healthy Eating Index-2015 (HEI, range 0-100), calculated across pregnancy. Networks from 40 food groups were derived for women in the highest and lowest HEI tertiles at each participant-labeled meal (i.e., breakfast, lunch, dinner, snacks) using Gaussian graphical models. Network composition was qualitatively compared across meals and between HEI tertiles.

**Results:**

In both HEI tertiles, breakfast food combinations comprised ready-to-eat cereals with milk, quick breads with sweets (e.g., pancakes with syrup), and bread with cheese and meat. Vegetables were consumed at breakfast among women in the high HEI tertile only. Combinations at lunch and dinner were more varied, including vegetables with oils (e.g., salads) in the high tertile and sugary foods with nuts, fruits, and milk in the low tertile at lunch; and cooked grains with fats (e.g., pasta with oil) in the high tertile and potatoes with vegetables and meat in the low tertile at dinner. Fried potatoes, sugar-sweetened beverages, and sandwiches were consumed together at all main meals in the low tertile only. Foods were consumed individually at snacks in both tertiles; the most commonly consumed food were fruits in the high HEI tertile and cakes & cookies in the low tertile.

**Conclusions:**

In this cohort of pregnant women, food network analysis indicated that food combinations differed by meal and between HEI tertiles. Meal-specific patterns that differed between diet quality tertiles suggest potential targets to improve food choices at meals; the impact of meal-based dietary modifications on intake of correlated foods and on overall diet quality should be investigated in simulations and intervention studies.

**Trial registration:**

PEAS was registered with number NCT02217462 in Clinicaltrials.gov on August 13, 2014.

**Supplementary Information:**

The online version contains supplementary material available at 10.1186/s12966-021-01172-1.

## Introduction

Dietary intake among U.S. pregnant women typically exceeds recommendations for sodium [1, 2], empty calories [1], and total fat [3] and is below recommendations for whole grains [1, 2], fiber [4], vegetables [1–4], fruits [1, 2, 4], and dairy [1, 2]. Given associations of poor diet quality during pregnancy with numerous adverse maternal and child health outcomes [5–8], improving maternal diet quality is a public health priority [9, 10].

Overall diet quality results from hundreds of individual food choices [11–13] made within the context of individual eating occasions (i.e., meals) across days, and the amounts and composition of eating occasions vary considerably throughout the day [14]. Evidence suggests individuals consume foods within eating occasions in predictable, socially constructed patterns; for example, a main meal may follow a tripartite structure centered around meat accompanied by a staple (e.g., potatoes) and trimmings (e.g., vegetables), while a light meal may comprise pasta with sauce, and a snack may focus on portable items [15, 16]. However, most research investigating the contribution of individual eating occasions to overall diet quality has examined isolated meals or dietary factors, such as the significance of breakfast [17–19]. Findings from few studies investigating relations of overall diet intake with various aspects of meal-specific intake at multiple eating occasions show that differences in food choices at meals explain a large proportion of the variation in overall energy and macronutrient intake [20, 21], but little is known about their contribution to diet quality. Understanding meal-specific food combinations in pregnant women, and the relationships of these food combinations with overall diet quality may identify behavioral targets for improving overall diet quality.

Meal-specific dietary intake can be analyzed using Gaussian graphical model (GGM) derived food networks, representing probabilistic graphs that show the underlying relationship structure among food groups using partial correlations [22, 23]. This approach provides unique information that may not be captured by traditional methods. For example, principle component analysis is commonly used to derive meal-specific dietary patterns [24, 25] that can then be used in quantitative models. However, applied to meal data, PCA can result in dozens of patterns while explaining only a small proportion of the variability of food intake, and the resulting patterns do not provide information on the interrelationships among food groups. In contrast, GGM networks facilitate interpretation of dietary intake as a set of inter-dependent food groups and reveals patterns of food group combinations specific to each meal. The aim of this study was to utilize GGM to identify and qualitatively compare meal-specific food networks from pregnant women with high and low diet quality to better understand how meal food group composition contributes to overall diet quality during pregnancy.

## Subjects and methods

### Participants and recruitment

Women (*n* = 458) aged 18-44 years receiving prenatal care at the obstetrics clinics at the University of North Carolina (UNC) at Chapel Hill Healthcare System were enrolled in the Pregnancy Eating Attributes Study (PEAS) at ≤12 weeks gestation and assessed each pregnancy trimester [26]. Additional eligibility criteria included: anticipating uncomplicated singleton pregnancy, willing to undergo study procedures and provide informed consent for her participation and assent for the baby’s participation, BMI ≥ 18.5 kg/m^2^, ability to complete self-report assessments in English, access to internet with email, planning to deliver at UNC Hospital, and planning to remain in the geographical vicinity for 1 year following delivery. Exclusion criteria were pre-existing diabetes, multiple pregnancy, participant-reported eating disorder, and any medical or psychosocial condition contraindicating participation in the study. Additional study details are available elsewhere [26]. Data collection occurred from 2014 to 2018.

### Dietary assessment

Participants were asked to complete three 24-h recalls throughout pregnancy (one per trimester) using the web-based NCI Automated Self-Administered 24-Hour Dietary Assessment Tool [27], which prompts respondents to report all food and beverages consumed at each eating occasion over the preceding day (from awakening until going to bed). Respondents reported the time and type of eating occasion, which may include “breakfast”, “brunch”, “lunch”, “dinner”, “supper”, “snack”, “just a drink”, and “just a supplement” by order of occurrence. The USDA’s Food and Nutrient Database for Dietary Studies (FNDDS) [28] was used to compute nutrients, foods, and overall diet quality as per the Healthy Eating Index (HEI) 2015 scores. The HEI-2015, a multi-component diet quality score, was developed and validated to evaluate adherence to the 2015-2020 Dietary Guidelines for Americans (DGA) [29], which applies to Americans 2 years of age and older, including pregnant women [29]. The HEI-2015 consists of 9 adequacy components (total fruit, whole fruit, total vegetables, greens and beans, whole grains, dairy, total protein foods, seafood and plant proteins, and fatty acids) and 4 moderation components (refined grains, sodium, added sugars, and saturated fats), scored based on energy-adjusted food and nutrient intakes in order to enable comparisons across individuals with varying energy requirements [30, 31]. The component scores are summed to yield a total score with a maximum of 100 (higher scores indicate a healthier diet). Previous studies have reported little change in diet quality across pregnancy trimesters [32, 33], which is consistent with data from the same study population [34]; therefore, pregnancy diet recalls were pooled to calculate HEI across pregnancy using the simple HEI scoring algorithm – per person [35]. Implausible reporting of energy intake has previously been examined based on cutoffs of < 500 and > 3500 kcal in non-pregnant adults [36]. To account for increased energy requirements of pregnancy [37], this threshold was increased; individual food item data from dietary recalls indicating total energy intake < 600 kcal and > 4500 kcal were examined for implausibility by three members of the study team. All records with energy intake < 600 kcal were considered implausible and, therefore, excluded from analysis, while recalls with energy intake > 4500 kcal were considered to reflect plausible intake and were, therefore, retained for analyses.

### Assessment of anthropometric and demographic data

Maternal height was measured at baseline (≤12 weeks gestation) and weight was measured at baseline and each pregnancy trimester (13-18 weeks, 16-22 weeks, and 28-32 weeks gestation). Early pregnancy BMI (kg/m^2^) was calculated from measured height and weight at baseline. Gestational weight gain was calculated as the difference between weight at the last visit (0.35 ± 0.75 weeks before delivery) and baseline weight, and classified as inadequate, adequate, or excessive based on IOM guidelines for weekly range of weight gain [9]. Mothers reported sociodemographic data at baseline.

### Food intake modeling

Foods were grouped into 40 categories (Additional File 1) based on FNDDS categories. Mixed dishes were broken down into component foods when the breakdown provided further information about the healthfulness of the food or when the components themselves were a separate food group (e.g., FNDDS category “meat, poultry, fish in gravy or sauce or creamed” was separated into “meat”, “poultry”, or “fish”, and “sauce”). Previous research suggests that people conceptualize foods in terms of familiar dishes rather than their components [38] (e.g., pizza versus bread, cheese, and tomato sauce). Breaking these foods down to components may obscure relations of the mixed dishes to the other foods eaten with it [39]. Therefore, FNDDS mixed dishes were not broken down into components if this resulted in a substantial alteration of the conceptualization. These foods were categorized based on similarity of ingredients or type of food (e.g., meatloaf and crab cakes as protein-based patties and loaves).

Food intake for the FNDDS-based 40 food groups (in grams) at each meal was used to derive a food network for each meal (i.e., breakfast, lunch, dinner, and snacks) for each HEI tertile. Dinner networks included meals reported as supper. All meals were considered as independent observations to retain the meal structure and identify foods that were consumed together in a single eating occasion. Meal-specific food networks were obtained separately for the low and high HEI tertiles. To examine trimester-specific meal composition variations, trimester-specific meal networks for each HEI tertile were also derived based on dietary recalls provided during the first, second, and third trimesters.

### Statistical analysis

To compare groups clearly different in diet quality, participants were classified into HEI tertiles, and the low (*n* = 121) and high (*n* = 122) tertiles were used for analysis to reflect low and high diet quality, respectively. Networks were derived through GGM, which are probabilistic, undirected graphs describing conditional independence between variables. Resulting graphs are networks consisting of a set of nodes (i.e. food groups) and edges or lines between them (i.e. partial correlations), representing conditional dependence between food groups [22]. GGM networks are quantified using the inverse covariance matrix yielding partial correlations under the assumption of a normal distribution [40]. High-dimensional multivariate data can have no or few 0 values, which would produce a dense, less informative graph [22]. The aim of GGM is to achieve an accurate and meaningful representation of the underlying covariance structure of the data. The graphical lasso method estimates a sparse or regularized partial correlation matrix – where zeros correspond to pairs of conditionally independent variables – by setting a threshold on the off-diagonal elements of the inverse covariance matrix, shrinking the estimated partial correlations and avoiding overfitting of the model (i.e. false inclusion of edges) [41]. The degree of regularization or sparsity is set by the penalty parameter lambda (λ >  0) and depends on the research question and model fit [22]. For this study, the penalty parameter was determined by selecting the optimal lambda from a 5-fold cross-validated graphical lasso. Because meal-level dietary data are non-normally distributed (based on visual inspection of the histograms), the semiparametric Gaussian copula graphical model (SGCGM), a semiparametric extension of GGM, was used. This approach uses Spearman’s rho and Kendall’s tau to estimate the correlation matrix, which is then entered into the parametric procedure – the graphical lasso – to obtain the final estimate of the regularized partial correlation matrix [42, 43]. Additionally, to address the high proportion of zeros and avoid overrepresentation of relationships between foods consumed episodically (i.e., not all 40 food groups were consumed in each meal, therefore many food groups within meals have intakes of 0 g), different constraints were tested, resulting in exclusion of foods consumed on fewer than 5% of meals per network. Additional File 2 shows the network and community properties of the different constraints that were tested, including optimal lambdas obtained from 5-fold cross-validated graphical lasso used in the analyses. For the trimester-specific meal networks, the same optimal lambda from the respective meal and a food group threshold of 5% of meals was used.

Communities comprised of at least two food groups subdivide networks into smaller combinations of foods that are more densely connected to each other than to foods in the rest of the network. Communities were detected based on the absolute value of the partial correlations using the Louvain method (LM), frequently used for analyzing large weighted networks [44, 45]. After community detection, we classified nodes into roles according to their intra- and inter-community connectivity pattern measured by the within-community degree (WC) and the participation coefficient (PC) using the method from Guimerà et al. [46]. The WC measures how well a node connects to the rest of its community and is expressed as a z-score, where a higher score indicates a higher internal connectivity relative to its community. The PC measures the degree of connectivity with other communities relative to the connections within its own community, with a value of 0 if all edges are within its community and close to 1 if edges are uniformly distributed among communities [46]. We adapted the WC threshold to fit our data: nodes with WC greater than or equal to 1.0 were classified as hubs and nodes with WC lower than 1.0 as non-hubs. Nodes were further classified according to their PC: hub nodes were divided into provincial hubs (i.e., food groups having most edges within rather than external to their community), connector hubs (i.e., food groups having multiple edges with other communities), and kinless hubs (i.e., food groups having edges uniformly distributed among communities) [46]. Non-hub nodes were divided into ultra-peripheral nodes (i.e., food groups having all edges within rather than external to their community), peripheral nodes (i.e., food groups having most edges within their community), non-hub connector nodes (i.e., food groups having multiple edges with other communities), and non-hub kinless nodes (i.e., food groups having uniformly distributed among communities) [46] (Table 1). While provincial hubs have an important structural role within their community, connector nodes contribute to higher connectivity between communities (i.e., greater network integration) [47]. Food networks were evaluated qualitatively by describing and comparing network, community, and node role (hub and connector nodes) between HEI tertiles (Additional File 3). For the purpose of identification, communities were numbered arbitrarily; communities composed of similar food combinations in both HEI tertiles were assigned the same number.

**Table 1:** Node role classification used in food networks^a^

Node role classification	WC^b^	PC	Description
Provincial hub	≥ 1.0	≤ 0.30	Most edges within its own community
Connector hub	≥ 1.0	> 0.3 and ≤ 0.75	Many edges to other communities
Kinless hub	≥ 1.0	> 0.75	Edges uniformly distributed among other communities
Ultra-peripheral	< 1.0	≤ 0.05	All edges within its community
Peripheral	< 1.0	> 0.05 and ≤ 0.62	Most edges within its community
Non-hub connector	< 1.0	> 0.62 and ≤ 0.80	Many edges to other communities
Non-hub kinless	< 1.0	≥ 0.80	Edges uniformly distributed among other communities

*PC* participation coefficient, *WC* within-community degree

^a^ Using the method from Guimerà et al. [46]

^b^ WC threshold adapted to fit network sparsity of our data

The intra-class correlation (ICC) was calculated to assess inter-individual and inter-meal variation of each food group. ICC was calculated based on the multi-level approach of Bell, et al. [48] in: 1) a three level model for inter-meal variation (highest level of the data corresponding to participants, next level corresponding to type of meal – breakfast/lunch/dinner/snacks; ICC for type of meal indicates variation in food intake explained by type of meal); and 2) a two level model for inter-individual variation at specific meals (highest level of the data corresponding to participants; ICC indicates variation in food intake explained by differences between participants at each meal).

Data management was conducted in SAS (Version 9.4, Enterprise Guide 7.1, SAS Institute Inc., Cary, NC, USA) and network analysis was performed in R (Version 3.6.1, R Foundation for Statistical Computing, Vienna, Austria). R package *huge* [49] was used for data transformation, *nethet* [50] for cross-validated graphical lasso (to find optimal lambda), *glasso* to obtain food networks [51], and *NetworkToolbox* [52] for Louvain community detection. Cytoscape, version 3.7.2 [53], was used for data visualization.

## Results

**Fig. 1:**
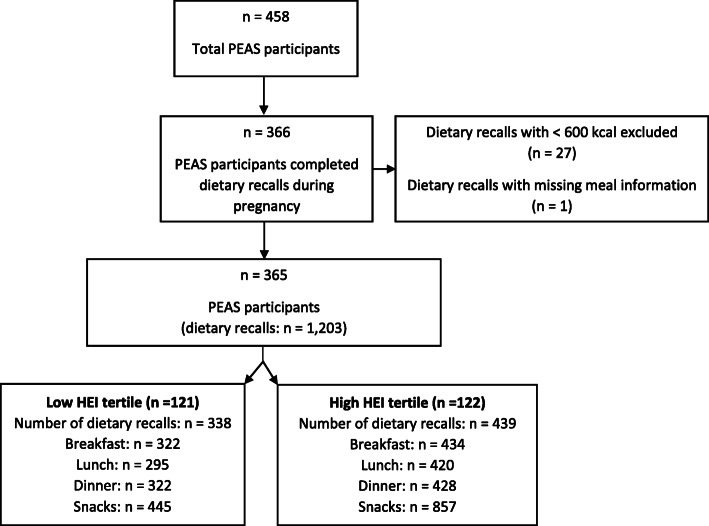
Flow diagram of PEAS participants for analysis in the present study

Of 458 women enrolled, 366 completed dietary recalls during pregnancy. After excluding dietary recalls < 600 kcal and one with missing meal information, dietary data from 365 women were available. Data from 121 participants in the low and 122 participants high HEI tertiles were used for meal food network analysis; participants in the high HEI tertile completed more dietary recalls and consumed more snacks per day than participants in the low HEI tertile (Fig. 1). Participants in the high HEI tertile were on average older, had more often normal weight and higher education level, were more often white, employed full time, and most were married and/or living with their partner (Table 2).

**Table 2:** Sample characteristics of the analytic sample by HEI tertile^a^

Characteristics	HEI tertile	
	Low (*n* = 121)	High (*n* = 122)
Age (years), mean (SD)	29.7 (5.3)	31.5 (3.6)
BMI (kg/m^2^)		
Normal (≥ 18.5 to < 25)	50 (41.3)	75 (61.5)
Overweight (25 to < 30)	33 (27.3)	33 (27.1)
Obese (≥ 30)	38 (31.4)	14 (11.5)
Race Ethnicity		
White	71 (64.0)	88 (75.2)
Black	24 (21.6)	8 (6.8)
Hispanic or Latino	11 (9.9)	8 (6.8)
Other	5 (4.5)	13 (11.1)
Education (highest degree obtained)		
Some college or less	51 (46.8)	11 (9.6)
Bachelor’s Degree	28 (25.7)	41 (36.0)
Graduate Degree	30 (27.5)	62 (54.4)
Employment status		
Full time	61 (56.0)	79 (69.3)
Part time	17 (15.6)	16 (14.0)
Not working	31 (28.4)	19 (16.7)
Marital status		
Married and/or living with partner	92 (84.4)	110 (96.5)
Other (divorced, widowed, separated, single)	17 (15.6)	4 (3.5)

*BMI* Body Mass Index, *HEI* Healthy Eating Index-2015

^a^ Values are frequency (%) unless otherwise indicated

**Fig. 2:**
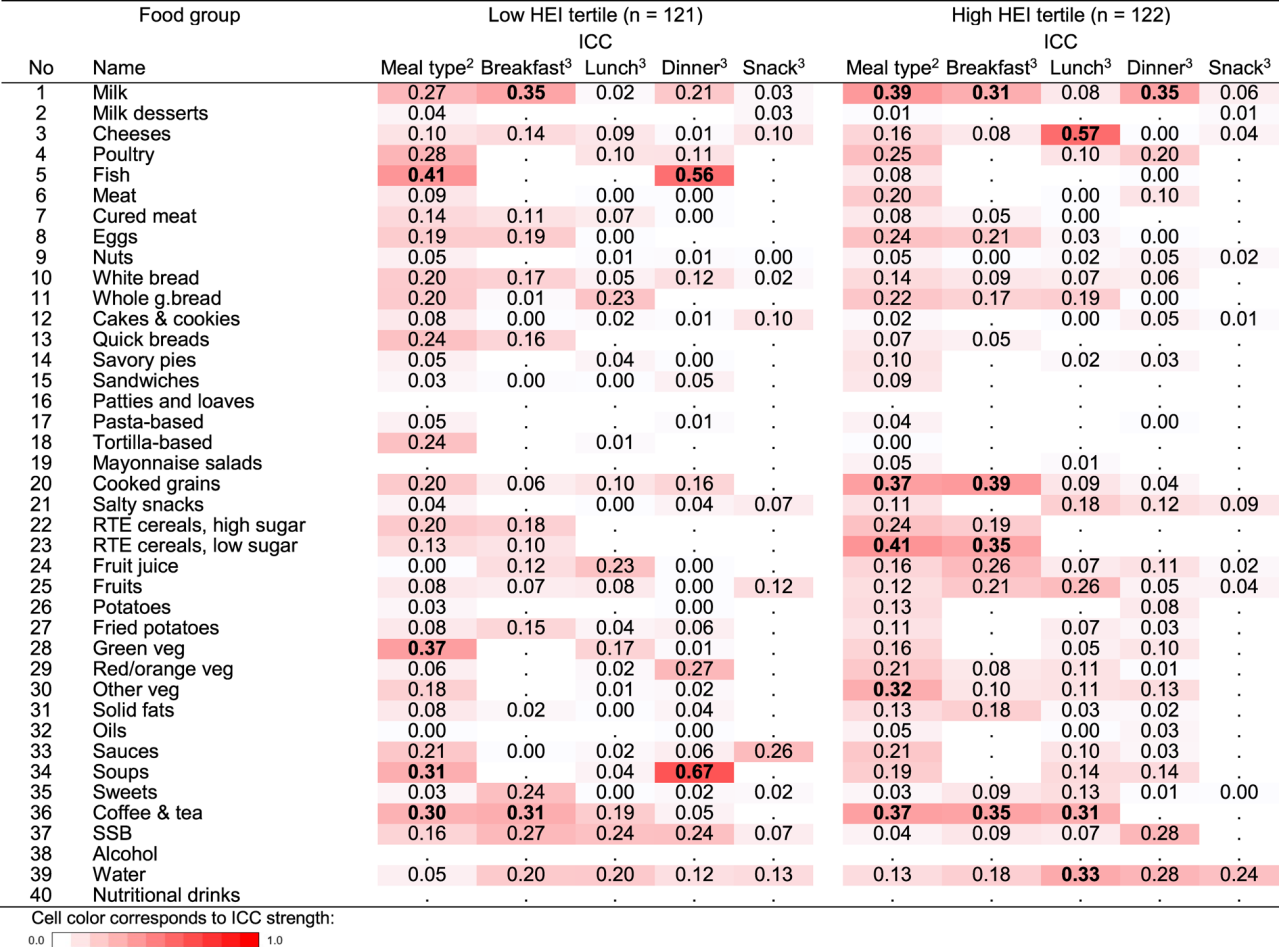
Heatmap of inter-meal and inter-individual variation in food intake (*n* = 243)^1^. HEI: Healthy Eating Index-2015; ICC: Intra-class correlation; RTE: ready-to-eat; SSB: sugar-sweetened beverages. ICCs of > 0.30 are marked in bold. ^1^ Intra-class correlation is presented for food groups consumed in at least 5% of the modelled recalls/meals by HEI tertile. ^2^ Variance explained by type of meal, considering inter-individual variation. ^3^ Variance explained by inter-individual variation for each meal type separately

Intraclass correlation coefficients indicated that in the low HEI tertile, between-meal variability (variability between meal type: breakfast vs. lunch vs. dinner vs. snacks) explained over 30% of the variation in intake of *fish* (higher at dinner), *green vegetables* (higher at lunch and dinner), *soups* (higher at lunch and dinner), and *coffee & tea* (higher at breakfast); in the high HEI tertile, between-meal variability explained over 30% of the variation in intake of *low-sugar ready-to-eat cereals* (higher at breakfast), *milk* (higher at breakfast), *cooked grains* (higher at breakfast and dinner), and *coffee & tea* (higher at breakfast) (Fig. 2).

### Breakfast networks

**Fig. 3:**
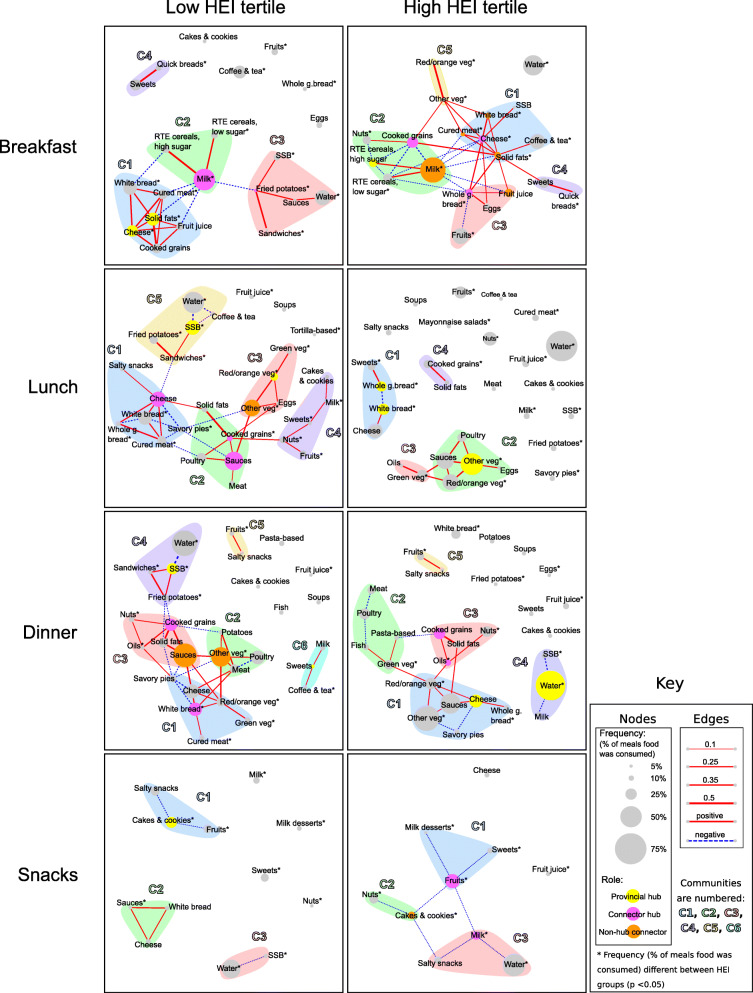
Meal food networks among PEAS participants in the low and the high HEI tertiles. HEI: Healthy Eating Index-2015. The thickness of the edges is proportional to the strength of the correlation. Blue, dashed edges indicate negative correlations and red, continuous edges indicate positive correlations. The size of the nodes is proportional to the percentage of meals in which the food was consumed and node role is indicated by colors yellow (provincial hub), purple (connector hub), and orange (non-hub connector). Communities are shown in different background colors around the nodes and are labeled C1-C6. An asterisk (*) next to food group labels indicates significant difference in node size by HEI tertile (*p* < 0.05), determined by Chi-square test

In the low HEI tertile (Fig. 3), nodes representing most commonly consumed food groups at breakfast included *milk* (51% of breakfasts), *water* (36%), and *white bread* (31%). Vegetables were not represented in any nodes. Four communities were identified, with two provincial hubs (*solid fats* and *cheese*) connecting all food groups within their community, and two connector hubs (*milk* and *fried potatoes*) connecting communities 1-3 exclusively through negative correlations, suggesting that participants consumed meals consisting of foods from single communities. *Milk* and *ready-to-eat cereals* (community 2) were positively correlated (i.e., consumed together), as were *sandwiches*, *sauces*, *sugar-sweetened beverages* (*SSB*)*,* and *fried potatoes* (community 3); and *quick breads* and *sweets* (community 4). In contrast, for five food groups, (*fruits*, *whole grain bread, coffee & tea, cakes & cookies,* and *eggs*), the absence of edges (correlations) to other food groups indicated conditional independence.

In the high HEI tertile (Fig. 3), nodes representing most commonly consumed food groups at breakfast included *milk* (64% of breakfasts), *water* (51%), *fruits* (40%), and *coffee & tea* (37%). Five communities were identified, with one provincial hub (*high-sugar ready-to-eat cereals*) connecting all food groups within its community. Multiple connector hubs (*cheese*, *cooked grains*, and *whole grain bread*) and non-hub connector nodes (*cured meat, solid fats, fruit juice, milk*, and *white bread*) indicate high connectivity between communities (i.e., greater network integration). All communities had positive correlations to other communities, suggesting that participants consumed meals consisting of foods from multiple communities. Communities 1, 2 and 4 were similar to those in the low HEI tertile, but community 1 included *SSB* and *coffee & tea* and community 2 included *cooked grains* (e.g., oatmeal) and *nuts*. *Red/orange vegetables* and *other vegetables* were positively correlated (community 5)*,* while *fruits* and *whole grain bread* were negatively correlated (community 3).

Intraclass correlation coefficients indicated that in the low HEI tertile, between-subject variability explained over 30% of the variation in intake of *milk* and *coffee & tea* (Fig. 2), indicating that these nodes may not represent intake patterns of all participants in the tertile. Similarly, in the high HEI tertile, between-subject variability explained over 30% of the variation in intake of *milk, coffee & tea, cooked grains*, and *low-sugar ready-to-eat cereals.*

### Lunch networks

In the low HEI tertile (Fig. 3), nodes representing most commonly consumed food groups at lunch included *water* (50% of meals), *sauces* (43%), *other vegetables* (37%), *cheese* (34%), *white bread* (32%), and *SSB* (32%). Five communities were identified, with two provincial hubs (*red/orange vegetables* and *SSB*) connecting all or most food groups within their communities. Multiple connector hubs (*cheese*, *sauces,* and *cooked grains*) and a non-hub connector node (*other vegetables*) indicate high connectivity between communities. While communities 1-4 shared at least one positive correlation, the only correlation connecting community 5 to another community was a negative correlation (*cheese* with *sandwiches*), suggesting that a single lunch meal may consist of community 5 foods only (e.g. *sandwiches*, *fried potatoes*, and *SSB*), or of combinations of foods from communities 1-4.

In the high HEI tertile (Fig. 3), nodes representing most commonly consumed food groups at lunch included *water* (71% of meals), *other vegetables* (49%), *sauces* (37%), *red/orange vegetables* (35%),and *cheese* (32%). Four communities were identified, with three provincial hubs (*whole grain bread, white bread*, and *other vegetables*) connecting all or most food groups within their communities, and with no connector hubs or non-hub connector nodes, indicating low connectivity between communities and suggesting that participants consumed meals consisting of foods from single communities. Communities 2 and 3 included food groups similar to those in the low HEI tertile, but with *cooked grains* and *solid fats* constituting their own community (community 4). In the high HEI tertile, *sauces* were also positively correlated with *poultry* and vegetables, but not with *cooked grains* or *meat*. For several food groups (*fruits, coffee & tea, soups, salty snacks, mayonnaise salads, cured meat, nuts, fruit juice, water, meat, cakes & cookies, milk, SSB, fried potatoes,* and *savory pies*), the absence of edges (correlations) to other food groups indicated conditional independence.

In the high HEI tertile only, between-subject variability explained over 30% of the variation in intake of some foods (*cheese*, *water*, and *coffee & tea*) (Fig. 2), indicating that these nodes may not represent intake patterns of all participants in the tertile.

### Dinner networks

In the low HEI tertile (Fig. 3), nodes representing most commonly consumed food groups at dinner included *water* (57% of meals), *sauces* (52%), and other *vegetables* (42%). Six communities were identified, with two provincial hubs (*SSB* and *sweets*) connecting all food groups within their communities. Two connector hubs (*white bread* and *cooked grains*) connecting communities 1-4, and two non-hub connectors (*sauces* and *other vegetables*) connecting communities 1-3 indicate higher connectivity between communities. Strong positive correlations between communities 1-3 suggest that foods from these communities were consumed as part of the same meal, while communities 4-6 were conditionally independent of each other. *SSB, fried potatoes* and *sandwiches* were positively correlated with each other, and *SSB* was negatively correlated with *water* (community 4). For five food groups (*pasta-based dishes, fruit juice, cakes & cookies, fish,* and *soups*), the absence of edges (correlations) to other food groups indicated conditional independence.

In the high HEI tertile (Fig. 3), nodes representing most commonly consumed food groups at dinner included *water* (71% of meals), *other vegetables* (61%), and *sauces* (48%). Five communities were identified, with two provincial hubs (*cheese* and *water*) connecting all or most food groups within their communities. Fewer connector hubs (*cooked grains* and *oils*) and no non-hub connector nodes indicate lower connectivity between communities. Community 4 consists of only beverages, with *water* most frequently consumed and correlating negatively with *SSB* and *milk*. Community 5 was equivalent to its counterpart in the low HEI tertile, with a positive correlation between *fruits* and *salty snacks*. For eight food groups (*white bread, potatoes, soups, eggs*, *fried potatoes, fruit juice*, *sweets*, and *cakes & cookies*), the absence of edges (correlations) to other food groups indicated conditional independence.

In the low HEI tertile, between-subject variability explained over 30% of the variation in intake of *soups* and *fish* (Fig. 2), indicating that these nodes may not represent intake patterns of all participants in the tertile. Similarly, in the high HEI tertile, between-subject variability explained over 30% of the variation in intake of *milk*.

### Snacks networks

In the low HEI tertile (Fig. 3), nodes representing most commonly consumed food groups at snacks included *water* (41% of snacks), *cakes & cookies* (23%), and *salty snacks* (18%). Three conditionally independent communities were identified, with one provincial hub (*cakes & cookies*) at the center of community 1. While community 2 shows that *white bread*, *cheese*, and *sauces* (including condiments) were positively correlated, negative or no correlations in the rest of the network suggest that most foods were consumed alone.

In the high HEI tertile (Fig. 3), nodes representing most commonly consumed food groups at snacks included *water* (54% of meals), *fruits* (32%), and *nuts* (19%). Three communities were identified, with two connector hubs (*fruits* and *milk*) and one non-hub connector (*cakes & cookies*) connecting all communities through negative correlations. Like the low HEI tertile snack network, negative or no correlations suggest that foods were consumed alone.

Intraclass correlation coefficients indicated that between-subject variability explained less than 30% of the variation in intake of all food groups in both HEI tertiles (Fig. 2), indicating that nodes roughly represent intake patterns of every participant in each tertile.

### Trimester-specific meal networks

Overall, minor differences were found in meal networks across pregnancy trimesters; nevertheless, the overall structure remained. At main meals (i.e., breakfast, lunch, dinner), the second and third trimester networks were more integrated, in which case higher density of connections and fewer conditionally independent food groups produced somewhat altered community composition, but most of the original connections still remained (results not shown).

## Discussion

GGM-derived food networks are a novel analytic approach that facilitates investigation of diet from the perspective of the inter-dependence of food items within meal occasions rather than as summarizing over multiple meals and days. To our knowledge, this is the first study to examine associations between foods consumed within meals during pregnancy. Differences in meal patterns between diet quality tertiles varied across meals and were mostly consistent with overall diet quality classification, shown by differences in node size (indicating percentage of meals in which a food group was consumed), hub and connector nodes (indicating structurally important food groups), and combinations of food groups within communities. For example, node size was larger for *whole grain bread* in the high diet quality tertile and larger for *white bread* in the low diet quality tertile at breakfast. Similarly, *fried potatoes* and *SSB* acted as hubs for the low tertile but not for the high tertile, while *whole grain bread* was a hub in the high but not the low tertile.

These findings indicated several shared characteristics of meal-specific food group networks across diet quality tertiles. At breakfast, food group communities comprised of *white bread*, *cereals* and *milk*, and *quick breads* were observed in both diet quality tertiles. Also in both tertiles, foods at snacks were mostly consumed alone, rather than in combinations. Positive correlations were observed in both diet quality tertiles between two or more less healthful foods (i.e. foods contributing to a lower diet quality) like *sweets* and *quick breads* at breakfast in both tertiles, between two or more healthful foods (e.g., *green vegetables* and *red/orange vegetables* and *nuts* and *oils* at dinner in the low and the high diet quality tertile, respectively), as well as between more healthful and less healthful foods (e.g., *fruits* and *salty snacks* at dinner in both diet quality tertiles). Food networks were more integrated at breakfast, but less integrated at lunch and dinner in the high versus the low HEI tertile, suggesting the complexity of food combinations (e.g., number of food groups, number of possible different combinations) is not necessarily associated with diet quality, consistent with previous findings [15, 54]. The small differences found in trimester-specific meal networks could be partly randomly produced by the sample breakdown (trimester-specific networks have sample sizes ranging from 63 to 176 eating occasions, with a higher number of observations in the first trimester), but differences in network integration (lower in the first pregnancy trimester) could reflect dietary changes triggered by pregnancy symptoms, for example nausea and cravings in early and in mid-late pregnancy, respectively. Future studies should examine whether this is a persistent observation and whether pregnancy symptoms play a role.

Due to substantive differences in research questions and methods used, our findings cannot be directly compared with previous work on diet quality and meal composition during pregnancy. Studies examining dietary patterns in pregnancy are typically based on dietary assessments using FFQs [55], which preclude meal-specific analysis. However, our findings share some similarities with those from studies examining daily prenatal dietary intake in the U.S. [56, 57]. In a sample of predominantly lower- to middle-income pregnant women from North Carolina, French fries and soft drinks were among the top contributing foods to total energy and macronutrient intake and carbohydrate intake, respectively, suggesting these were commonly-consumed foods [56]. In our study, *SSB* and *fried potatoes* were most commonly consumed in the low diet quality tertile at lunch, suggesting a potential intervention target. In a sample of low-income pregnant women in Texas, frequent fast-food consumers ate more vegetables but also more gravy and less fruit [57]. Similar findings were observed in the current study among participants in the low diet quality tertile, in which *sandwiches*, *fried potatoes*, and *SSB*, which are often obtained from fast food outlets, were more frequently consumed, while *fruits* and *vegetables* were less frequently consumed, than in the high diet quality tertile.

Findings may inform potential guidance for addressing poor diet quality in this population. For example, one approach may include recommending the addition of vegetables to breakfasts. In the high diet quality tertile, cheese at breakfast was consumed with vegetables, eggs, and whole grain bread, which could be accomplished with a vegetable-filled omelet served with whole grain bread. The finding that foods were mostly consumed independently (i.e., not related to other food groups) at the snack eating occasions suggests that food intake at snacks may be more flexible than other meals, and therefore dietary changes at snacks may be easier to implement. However, the impact of meal-specific food intake changes on overall diet quality should be further investigated and confirmed in simulation and intervention studies. For example, studies on interventions aiming to reduce *white bread* intake at breakfast should assess the impact on other food groups such as *cured meat* and *high-sugar ready-to-eat cereals* as well as on overall diet quality. Some observed food combinations in the networks suggest that meal context may play an important role. For instance, the combination of *fried potatoes, sandwiches*, and *SSB* observed in all meal networks in the low diet quality tertile appears characteristic of a restaurant or fast-food meal pattern. Because food choices are affected by meal context [58, 59], recommendations aimed at changing food choices could have low adherence if not framed accordingly.

Strengths of this study include the large sample size and number of meal occasions, and the use of 24-h dietary recalls, supporting internal validity. Although no dietary assessment method is free of measurement error, 24-h recalls are considered to be the least biased self-report method, capturing diet with greater precision and detail than other methods such as food frequency questionnaires [60]. However, the generalizability of these findings is limited given the sample demographics. The skewed distribution with a high proportion of zeros is a limitation of the application of GGM-derived food networks to meal-specific dietary data, which we addressed by using a semiparametric extension of GGM, SGCGMs, and by excluding food groups consumed in fewer than 5% of the meals to avoid overrepresentation of the relationship between episodically-consumed foods eaten together on only a few occasions. Another consideration is that the correlations for the food networks in the high HEI tertile were based on a larger number of meals and dietary recalls. Furthermore, analyzing meals as independent observations retains the meal structure needed for this meal-specific analysis but does not account for repeated recalls from the same participant. However, most food groups had a low intra-class correlation in complementary analyses, suggesting low between-participant variation. On the contrary, foods with high intra-class correlation should be interpreted cautiously, such as *milk* and *coffee & tea* at breakfast, whose intakes were more unequally distributed between participants, with milk consumers and milk non-consumers. Finally, LM community detection provided valuable information about the structure of food combinations observed in the networks, but by visually emphasizing within-community correlations, important between-community correlations may be overlooked. Therefore, it is important to consider the entire network and not only the community break-down, especially in the presence of strong between-community correlations. Node role identification, specifically of connector nodes, addresses this issue and facilitates network interpretation.

## Conclusions

These findings regarding maternal meal-based dietary patterns may inform efforts to address overall poor diet quality among pregnant women. This GGM-derived meal food network analysis identified several differences in meal-specific dietary intake between pregnant women with low and high diet quality, including intake of vegetables, whole grain bread, cooked grains and nuts at breakfast and overall healthier snacks in the high diet quality group, and SSB, sandwiches and fried potatoes at all main meals – but most commonly at lunch – in the low diet quality group. Simulation studies and intervention studies are needed to test how changes in foods impact intake of correlated foods.

## Supplementary Information

**Additional file 1.**

**Additional file 2.**

**Additional file 3.**

**Additional file 4.**

## Data Availability

Data described in the manuscript, code book, and analytic code will be made available upon request pending approval of a data use agreement. Following publication of study objectives, de-identified data will be shared in the NICHD Data and Specimen Hub.

## Abbreviations

BMI: Body mass index
FNDDS: Food and Nutrient Database for Dietary Studies
GGM: Gaussian graphical models
HEI: Healthy Eating Index
LM: Louvain method
PEAS: Pregnancy Eating Attributes Study
SGCGM: Semiparametric Gaussian copula graphical models
SSB: Sugar-sweetened beverages
