# Supplementary material for: Using food network analysis to understand meal patterns in pregnant women with high and low diet quality
Source: Int J Behav Nutr Phys Act. 2021 Jul 23;18:101. doi: 10.1186/s12966-021-01172-1 (PMC8306349; doi:10.1186/s12966-021-01172-1)
Supplement: Supplementary file 1 — Additional file 1. [file 12966_2021_1172_MOESM1_ESM.docx]

ADDITIONAL FILE 1: Food categories used for network analysis

|  | Food group | Short name^1^ | Description, examples |
| --- | --- | --- | --- |
| 1 | Milk & milk drinks | Milk | Milk (regular, buttermilk, dry reconstituted, evaporated and condensed), yogurt, flavored milk and milk drinks, dry and powdered milk, sweet dairy cream and milk substitutes |
| 2 | Milk desserts | Milk desserts | Puddings, custards, and other milk desserts |
| 3 | Cheeses | Cheese | Natural cheeses, cottage cheeses, cream cheeses, processed cheeses and cheese spreads, imitation cheeses, cheese mixtures |
| 4 | Poultry | Poultry | Chicken, turkey, duck, other poultry |
| 5 | Fish & shellfish | Fish | Finfish, other seafood, shellfish |
| 6 | Meat | Meat | Beef, pork, lamb, veal, game |
| 7 | Cured & organ meat | Cured meat | Frankfurters, sausages, lunchmeats, meat spreads, organ meats and mixtures |
| 8 | Eggs | Eggs | Chicken eggs, other poultry eggs, egg substitute |
| 9 | Legumes, nuts & seeds | Nuts | Beans, peas, lentils, soybean products, meat substitutes (legume protein), nuts, nut butters, nut mixtures, seeds |
| 10 | Breads from refined grains | White bread | White bread rolls, biscuits, cornbread, corn muffins, tortillas, other quick breads |
| 11 | Whole grain breads | Whole g.bread | Whole wheat bread rolls, cracked wheat bread rolls, rye bread, oat bread, multigrain bread |
| 12 | Cakes, cookies, pies, pastries, bars | Cakes & cookies | Cakes, cookies, pies, cobblers, eclairs, turnovers and other pastries, Danish, doughnuts, coffee cake, cereal bars, nutrition bars |
| 13 | Pancakes, waffles, French toast, other grain products | Quick breads | Pancakes, waffles, French toast, crepes, rice flour cakes, funnel cakes |
| 14 | Savory pies and pastries | Savory pies | Egg rolls, spanakopitta, grape leaves stuffed with rice, quiche, turnovers, empanadas, puffs, tamales, pizzas and pizza rolls, calzone, won tons, dumplings, dim sum, pot pies |
| 15 | Sandwiches | Sandwiches | Sandwiches, wraps, and burgers |
| 16 | Protein-based patties and loaves | Patties & loaves | Meat loaf, meatballs, croquettes, crab cakes, salmon cakes/patties |
| 17 | Pasta-based mixed dishes | Pasta-based | Lasagna, cannelloni, ravioli, tortellini, macaroni and cheese, manicotti, stuffed shells |
| 18 | Tortilla-based mixed dishes | Tortilla-based | Burritos, chimichanga, tacos and taquitos, quesadillas, huevos rancheros |
| 19 | Mayonnaise-based salads | Mayonnaise salads | Egg salad and deviled eggs, chicken salad, tuna salad, shrimp salad, pasta salad with mayonnaise-type dressing |
| 20 | Pastas, rice, cooked cereals | Cooked grains | Pastas, rice, cooked cereals including oatmeal, grits, couscous, bulgur |
| 21 | Crackers & salty snacks from grain products | Salty snacks | Crackers, sweet crackers, low sodium crackers, non-sweet crackers, salty snacks from grain products |
| 22 | High-sugar ready-to-eat cereals | RTE cereals, high sugar | Ready-to-eat cereals, >21.2g/100g sugar |
| 23 | Low-sugar ready-to-eat cereals | RTE cereals, low sugar | Ready-to-eat cereals, <21.2g/100g, sugar |
| 24 | Fruit juices | Fruit juice | Fruit juices, nectars |
| 25 | Whole fruits | Fruits | Fresh fruits, mixtures of fruits, dried fruits |
| 26 | White potatoes & starchy vegetables | Potatoes | White potatoes, baked and boiled, creamed, scalloped, au gratin, mashed, stuffed, puffs, potato salad, Puerto Rican starchy vegetables |
| 27 | White potatoes, fried, chips & sticks | Fried potatoes | White potato chips and sticks, fried, hash browns |
| 28 | Dark green vegetables | Green veg | Dark green leafy vegetables, broccoli |
| 29 | Red & orange vegetables | Red/orange veg | Tomatoes, carrots, pumpkin, squash, sweet potatoes |
| 30 | Other vegetables | Other veg | Alfalfa sprouts, artichoke, asparagus, bamboo shoots, cauliflower, celery, corn, cucumber, eggplant, fennel, garlic, mushrooms, olives, onions, peppers, water chestnuts |
| 31 | Solid fats | Solid fats | Butter, cocoa butter, coconut oil, lard, shortening |
| 32 | Oils | Oils | Oil from nuts, seeds, olives, avocados, grains, and fish oil |
| 33 | Sauces, dressings & condiments | Sauces | White sauces and gravies, tomato-based sauces, soy sauce-based sauces, cream-based sauces, sour cream, salad dressings, ketchup, mayonnaise, mustard |
| 34 | Soups | Soups | Soups, broths, stews |
| 35 | Sugars & sweets | Sweets | Sugars, sugar replacements or substitute, syrups, honey, molasses, sweet toppings, jellies, jams, preserves, gelatin desserts, ices or popsicles, candies, chewing gums |
| 36 | Coffee & tea | Coffee & tea | Coffee, coffee substitutes, tea |
| 37 | Sugar-sweetened beverages (SBB) | SSB | Carbonated soft drinks, fruit drinks, non-fruit beverages, nonalcoholic beers, wines and cocktails, energy drinks |
| 38 | Alcoholic drinks | Alcohol | Beers and ales, liquors, cocktails, wines |
| 39 | Water, noncarbonated | Water | Tap, bottled |
| 40 | Nutritional drinks | Nutr. drinks | Meal replacement drinks, protein powder mixes |

^1^ Short name used in network labels, tables, and manuscript text
