# Supplementary material for: Using food network analysis to understand meal patterns in pregnant women with high and low diet quality
Source: Int J Behav Nutr Phys Act. 2021 Jul 23;18:101. doi: 10.1186/s12966-021-01172-1 (PMC8306349; doi:10.1186/s12966-021-01172-1)
Supplement: Supplementary file 2 — Additional file 2. [file 12966_2021_1172_MOESM2_ESM.docx]

ADDITIONAL FILE 2: Network and community properties with varying constraints for frequency of consumption

| Network, degree of constraint^1^ | Optimal Lambda (penalty parameter) | No. of food groups | No. of edges | Network edge density^2^ | Communities^3^ | Modularity | Clustering coefficient | |
| --- | --- | --- | --- | --- | --- | --- | --- | --- |
| Breakfast, HEI-L | |  |  | | | | |  |
| no constraint^4^ | 0.20 | 30 | 54 | 1.80 | 4 | 0.53 | 0.38 | |
| 5%^5^ | 0.22 | 21 | 24 | 1.14 | 4 | 0.44 | 0.19 | |
| 10% | 0.22 | 16 | 19 | 1.19 | 3 | 0.29 | 0.26 | |
| 15% | 0.23 | 11 | 16 | 1.45 | 2 | 0.15 | 0.32 | |
| Breakfast, HEI-H | |  |  | | | | |  |
| no constraint | 0.14 | 32 | 95 | 2.97 | 3 | 0.37 | 0.40 | |
| 5% | 0.17 | 20 | 37 | 1.85 | 5 | 0.31 | 0.38 | |
| 10% | 0.24 | 15 | 9 | 0.60 | 2 | 0.38 | 0.11 | |
| 15% | 0.23 | 12 | 5 | 0.42 | 2 | 0.33 | 0.19 | |
| Lunch, HEI-L | |  |  | | | | |  |
| no constraint | 0.12 | 35 | 87 | 2.49 | 4 | 0.41 | 0.23 | |
| 5% | 0.17 | 28 | 36 | 1.29 | 5 | 0.58 | 0.25 | |
| 10% | 0.19 | 16 | 17 | 1.06 | 4 | 0.58 | 0.36 | |
| 15% | 0.20 | 10 | 7 | 0.70 | 3 | 0.62 | 0.30 | |
| Lunch, HEI-H | |  |  | | | | |  |
| no constraint | 0.17 | 38 | 30 | 0.79 | 6 | 0.60 | 0.17 | |
| 5% | 0.23 | 28 | 13 | 0.46 | 4 | 0.48 | 0.10 | |
| 10% | 0.20 | 19 | 12 | 0.63 | 2 | 0.42 | 0.23 | |
| 15% | 0.18 | 13 | 12 | 0.92 | 2 | 0.37 | 0.35 | |
| Dinner, HEI-L | |  |  | | | | |  |
| no constraint | 0.18 | 38 | 38 | 1.00 | 6 | 0.57 | 0.18 | |
| 5% | 0.17 | 29 | 38 | 1.31 | 6 | 0.45 | 0.25 | |
| 10% | 0.16 | 16 | 29 | 1.81 | 4 | 0.31 | 0.36 | |
| 15% | 0.21 | 12 | 14 | 1.17 | 4 | 0.46 | 0.30 | |
| Dinner, HEI-H | |  |  | | | | |  |
| no constraint | 0.16 | 38 | 29 | 0.76 | 8 | 0.72 | 0.03 | |
| 5% | 0.15 | 28 | 22 | 0.79 | 5 | 0.57 | 0.04 | |
| 10% | 0.20 | 14 | 7 | 0.50 | 5 | 0.61 | 0.00 | |
| 15% | 0.14 | 12 | 10 | 0.83 | 3 | 0.33 | 0.15 | |
| Snacks, HEI-L | |  |  | | | | |  |
| no constraint | 0.17 | 27 | 24 | 0.89 | 5 | 0.41 | 0.20 | |
| 5% | 0.14 | 12 | 6 | 0.50 | 3 | 0.55 | 0.25 | |
| 10% | 0.16 | 6 | 1 | 0.17 | 1 | 0.00 | 0.00 | |
| 15% | 0.18 | 5 | 0 | 0.00 | 0 | 0.00 | 0.00 | |
| Snacks, HEI-H | |  |  | | | | |  |
| no constraint | 0.22 | 25 | 9 | 0.36 | 3 | 0.56 | 0.09 | |
| 5% | 0.12 | 10 | 8 | 0.80 | 3 | 0.26 | 0.00 | |
| 10% | 0.14 | 7 | 2 | 0.29 | 1 | 0.00 | 0.00 | |
| 15% | 0.16 | 6 | 1 | 0.17 | 1 | 0.00 | 0.00 | |

HEI: Healthy Eating Index-2015; HEI-L: low HEI tertile; HEI-H: high HEI tertile.

^1^ Excluding food groups that were consumed in less than 5%, 10%, or 15% of the occasions, respectively

^2^ Number of edges per food group

^3^ Louvain communities of at least 2 food groups

^4^ All food groups with at least one non-zero intake

^5^ Model used in all meal food networks.
