# Supplementary material for: Using food network analysis to understand meal patterns in pregnant women with high and low diet quality
Source: Int J Behav Nutr Phys Act. 2021 Jul 23;18:101. doi: 10.1186/s12966-021-01172-1 (PMC8306349; doi:10.1186/s12966-021-01172-1)
Supplement: Supplementary file 3 — Additional file 3. [file 12966_2021_1172_MOESM3_ESM.docx]

ADDITIONAL FILE 3: Node role classification in food networks

| **Node role classification^1^** | |  | **Breakfast networks** | |  | **Lunch networks** | |  | **Dinner networks** | |  | **Snacks networks** | |
| --- | --- | --- | --- | --- | --- | --- | --- | --- | --- | --- | --- | --- | --- |
|  |  |  | **HEI-L** | **HEI-H** |  | **HEI-L** | **HEI-H** |  | **HEI-L** | **HEI-H** |  | **HEI-L** | **HEI-H** |
| **Hub nodes**  **(WC > 1.0)** | **Provincial hubs (PC<=0.30)** |  | Cheese  Solid fats | RTE cereals, high sugar |  | Red/orange veg  SSB | White bread  Whole g.bread  Other veg |  | Sweets  SSB | Cheese  Water |  | Cakes & cookies |  |
|  | **Connector hubs (0.30<PC<=0.75)** |  | Milk  Fried potatoes | Cheese  Whole g.bread  Cooked grains |  | Cheese  Cooked grains  Sauces |  |  | White bread  Cooked grains | Cooked grains  Oils |  |  | Milk  Fruits |
| **Non-hub nodes**  **(WC < 1.0)** | **Ultra-peripheral**  **(PC <= 0.05)** |  | Quick breads  Sandwiches  Cooked grains  RTE cereals, low sugar  Sauces  Sweets  SSB  Water | Eggs  Nuts  Quick breads  Fruits  Red/orange veg  Sweets  Coffee &tea  SSB |  | Milk  Meat  White bread  Whole g.bread  Cakes & cookies  Salty snacks  Fruits  Fried potatoes  Green veg  Sweets  Coffee & tea  Water | Cheese  Poultry  Eggs  Cooked grains  Solid fats  Oils  Sweets |  | Milk  Poultry  Cured meat  Nuts  Sandwiches  Salty snacks  Fruits  Green veg  Coffee & tea  Water | Milk  Poultry  Fish  Meat  Nuts  Whole g.bread  Savory pies  Salty snacks  Fruits  Other veg  SSB |  | Cheese  White bread  Salty snacks  Fruits  Sauces  SSB  Water | Milk desserts  Nuts  Sweets  Water |
|  | **Peripheral (0.05<PC<=0.62)** |  | Cured meat  White bread  RTE cereals, high sugar  Fruit juice | RTE cereals, low sugar  Other veg |  | Poultry  Cured meat  Eggs  Nuts  Savory pies  Sandwiches  Solid fats | Green veg  Red/orange veg  Sauces |  | Cheese  Meat  Savory pies  Potatoes  Fried potatoes  Red/orange veg  Solid fats  Oils | Pasta-based  Green veg  Red/orange veg  Solid fats  Sauces |  |  | Salty snacks |
|  | **Non-hub connector (0.62<PC<=0.80)** |  |  | Milk  Cured meat  White bread  Fruit juice  Solid fats |  | Other vegs |  |  | Other veg  Sauces |  |  |  | Cakes & cookies |

HEI: Healthy Eating Index-2015; HEI-L: low HEI tertile; HEI-H: high HEI tertile.

PC: participation coefficient; WC: within-community degree.

1 No food groups were classified as kinless hubs or non-hub kinless.
